# Supplementary material for: Defining cellular complexity in human autosomal dominant polycystic kidney disease by multimodal single cell analysis
Source: Nat Commun. 2022 Oct 30;13:6497. doi: 10.1038/s41467-022-34255-z (PMC9618568; doi:10.1038/s41467-022-34255-z)
Supplement: Supplementary file 1 — Supplementary Information [file 41467_2022_34255_MOESM1_ESM.pdf]

Supplementary Information

Defining cellular complexity in human autosomal dominant polycystic kidney disease by multimodal single cell analysis

Yoshiharu Muto, Eryn E. Dixon, Yasuhiro Yoshimura, Haojia Wu, Kohei Omachi, Nicolas Ledru, Parker C. Wilson, Andrew J. King, N. Eric Olson, Marvin G. Gunawan, Jay J. Kuo, Jennifer Cox, Jeffrey H. Miner, Stephen L. Seliger, Owen M. Woodward, Paul A. Welling, Terry J. Watnick and Benjamin D. Humphreys

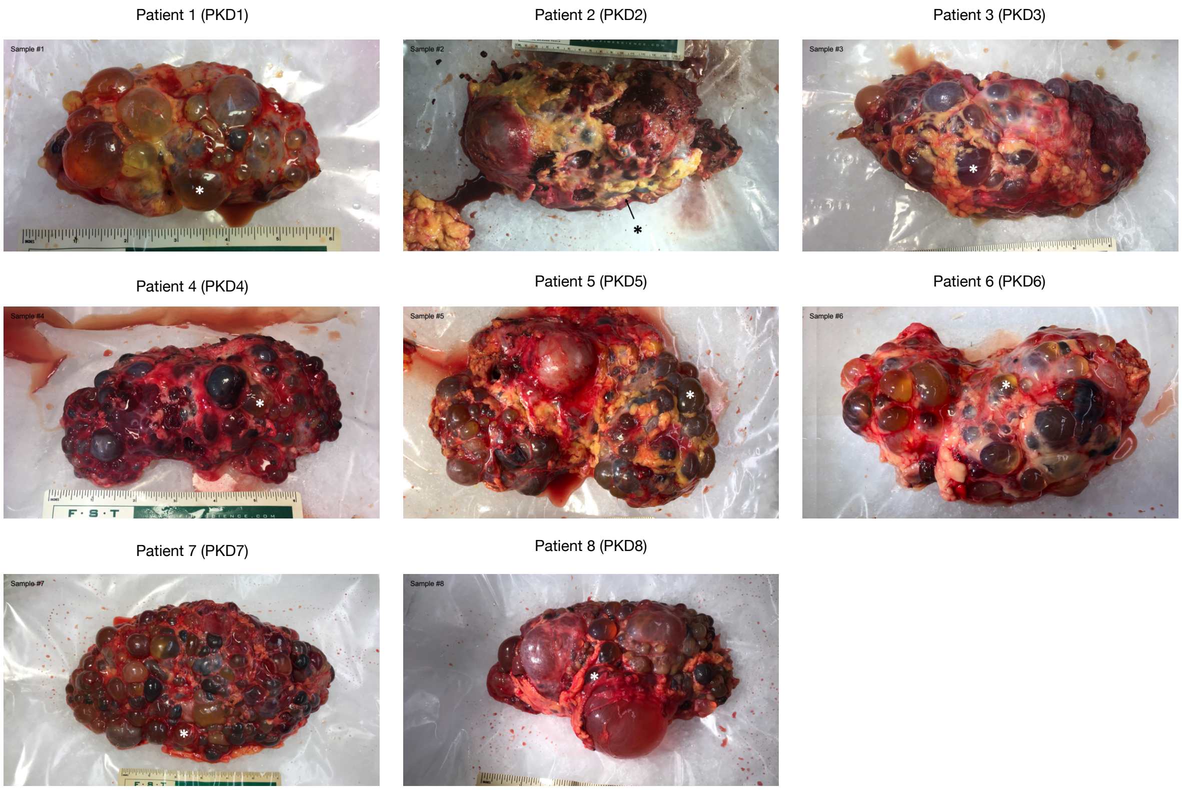

Supplementary Figure 1. Gross appearance of ADPKD kidney samples used for single cell analysis

ADPKD kidneys nephrectomized from ESKD patients. An asterisk (*) indicates the cyst from which a sample for single cell analysis was collected in each patient.

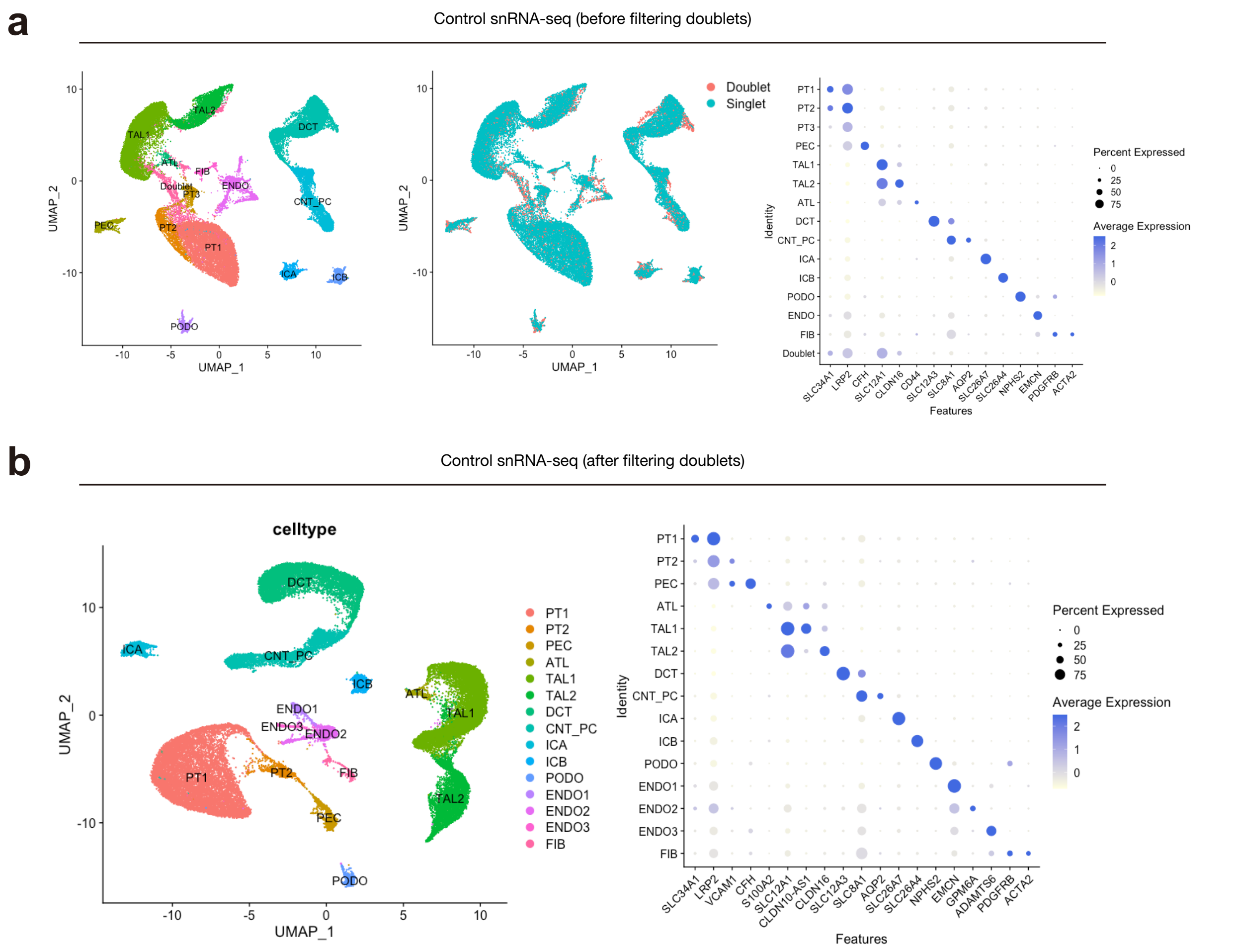

Supplementary Figure 2. Single cell transcriptional profiling on the control kidneys

(**a**) UMAP plots of control snRNA-seq dataset before filtering out doublets. Annotation by cell type (left) or doublet prediction by DoubletFinder (middle). Dot plot showing gene expression patterns of cluster-enriched markers (right). (**b**) UMAP plots of control snRNA-seq dataset after filtering out doublets. Annotation by cell type (left). Dot plot showing gene expression patterns of cluster-enriched markers (right). The diameter of the dot corresponds to the proportion of cells expressing the indicated gene and the density of the dot corresponds to average expression relative to all cell types.

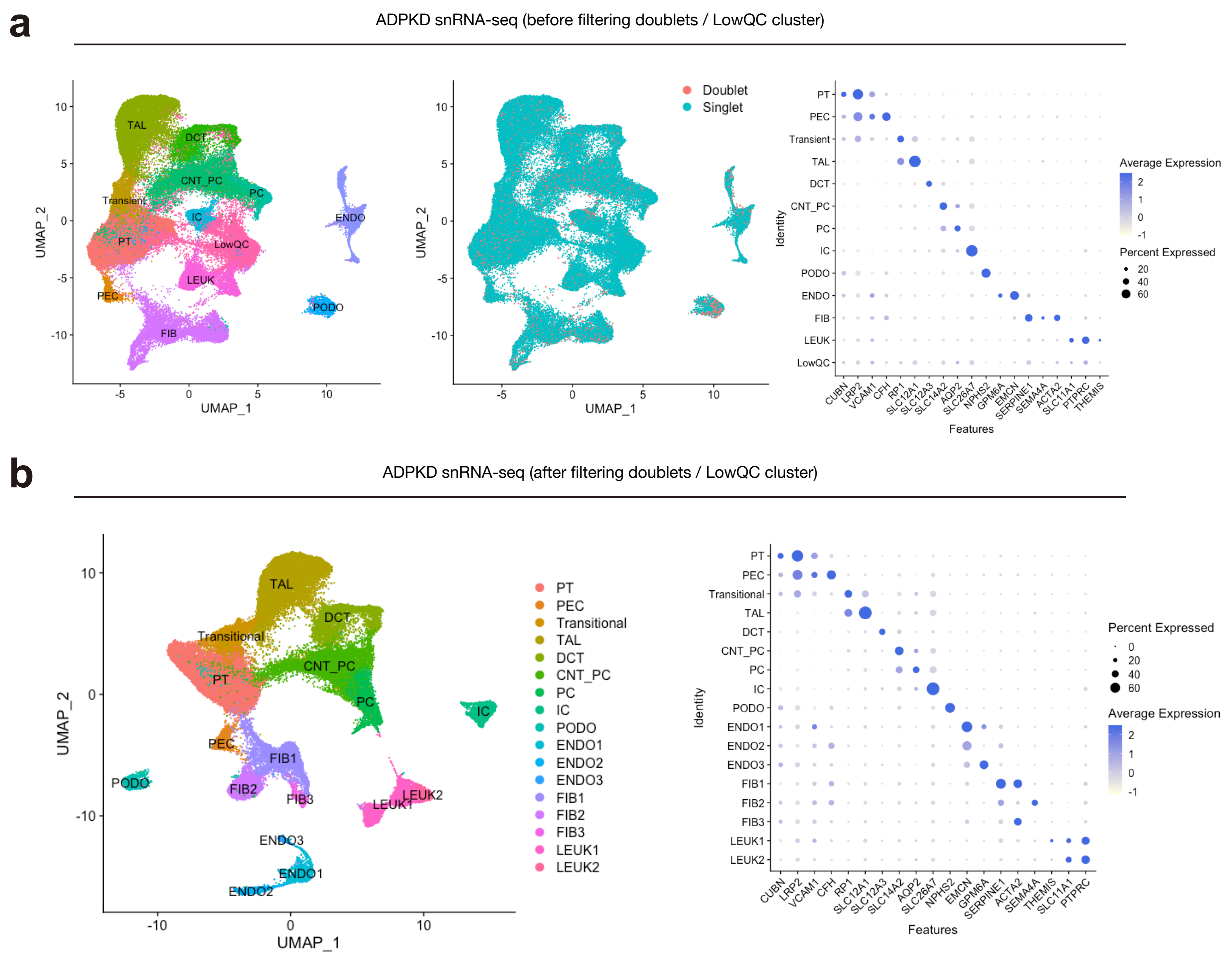

Supplementary Figure 3. Single cell transcriptional profiling on the ADPKD kidneys

(**a**) UMAP plots of ADPKD snRNA-seq dataset before filtering out doublets and low quality (LowQC) cluster. Annotation by cell type (left) or doublet prediction by DoubletFinder (middle). Dot plot showing gene expression patterns of cluster-enriched markers (right). (**b**) UMAP plots of ADPKD snRNA-seq dataset after filtering out doublets and LowQC cluster. Annotation by cell type (left). Dot plot showing gene expression patterns of cluster-enriched markers (right). The diameter of the dot corresponds to the proportion of cells expressing the indicated gene and the density of the dot corresponds to average expression relative to all cell types.

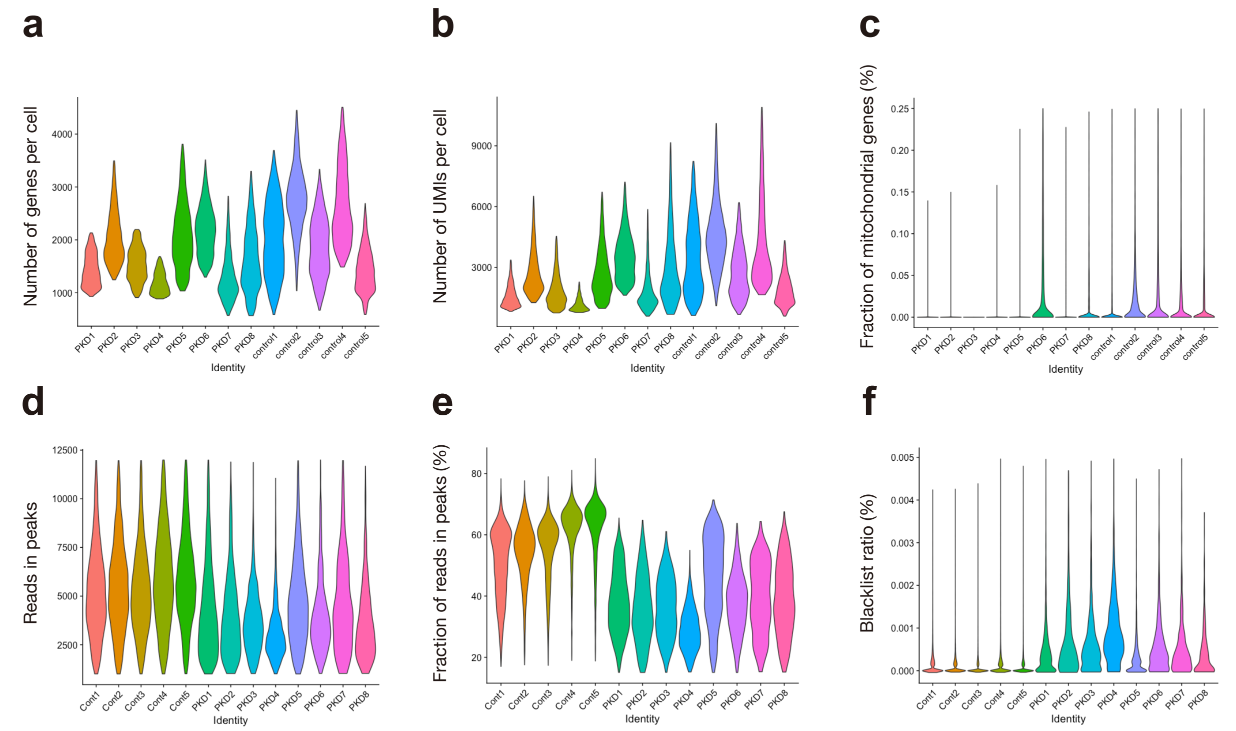

Supplementary Figure 4. QC metrics for snRNA-seq or snATAC-seq dataset: (**a**) Number of genes per cell, (**b**) number of UMIs per cell and (**c**) fraction of mitochondrial genes per cell in snRNA-seq data were shown. (**d**) Fraction of reads in peaks, (**e**) number of reads in peaks per cell and (**f**) ratio of reads in genomic blacklist region per cell in snATAC-seq data were shown.

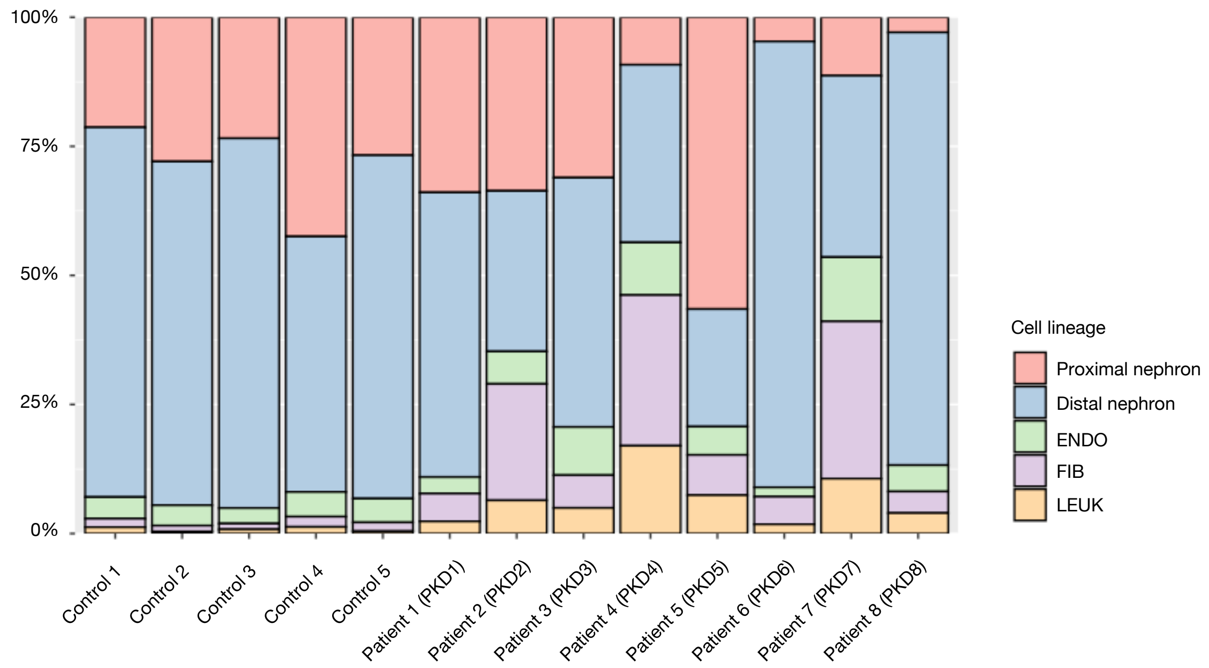

Supplementary Figure 5. Proportion of cell lineages in each dataset: The proportion of cell lineages in dataset from each sample in snRNA-seq. Proximal (PT, FR-PTC, PEC and PODO); Distal (TAL1, TAL2, DCT, CNT_PC and IC). Source data are provided as a Source Data file.

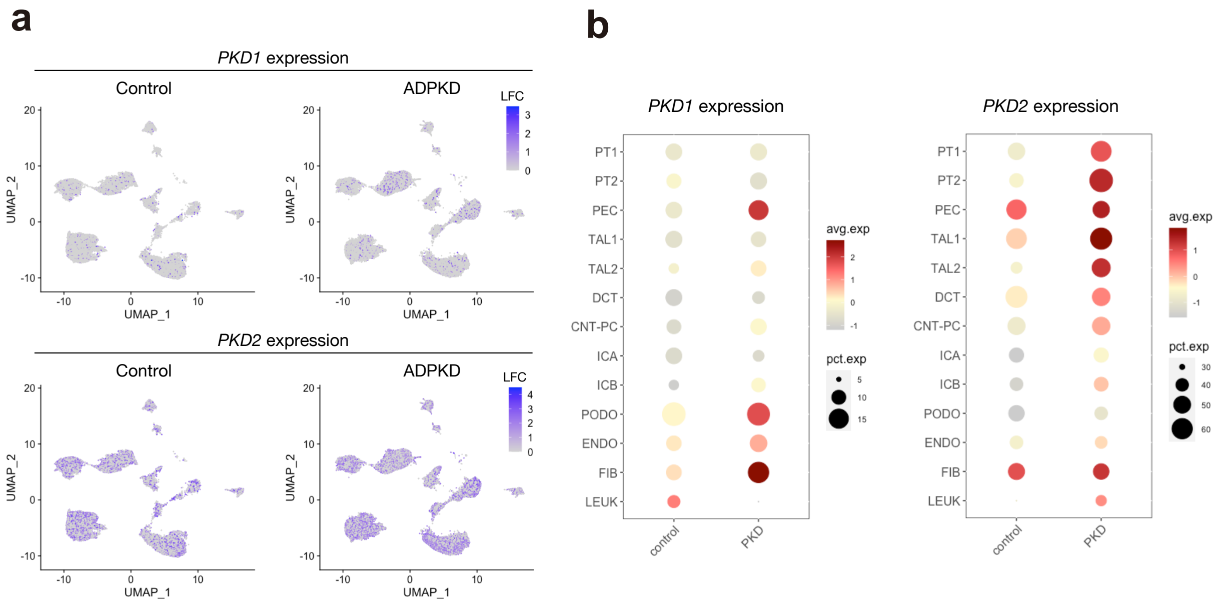

Supplementary Figure 6. *PKD1* or *PKD2* gene expressions in ADPKD dataset.

(**a**) UMAP plot displaying *PKD1* (upper panel) or *PKD2* (lower panel) gene expression in control or ADPKD dataset. The color scale for each plot represents a normalized log-fold-change (LFC). (**b**) Dot plot showing *PKD1* (left) or *PKD2* (right) gene expression in each celltype of control or ADPKD dataset. The diameter of the dot corresponds to the proportion of cells expressing the indicated gene (pct. exp) and the density of the dot corresponds to average expression relative to all celltypes (avg. exp).

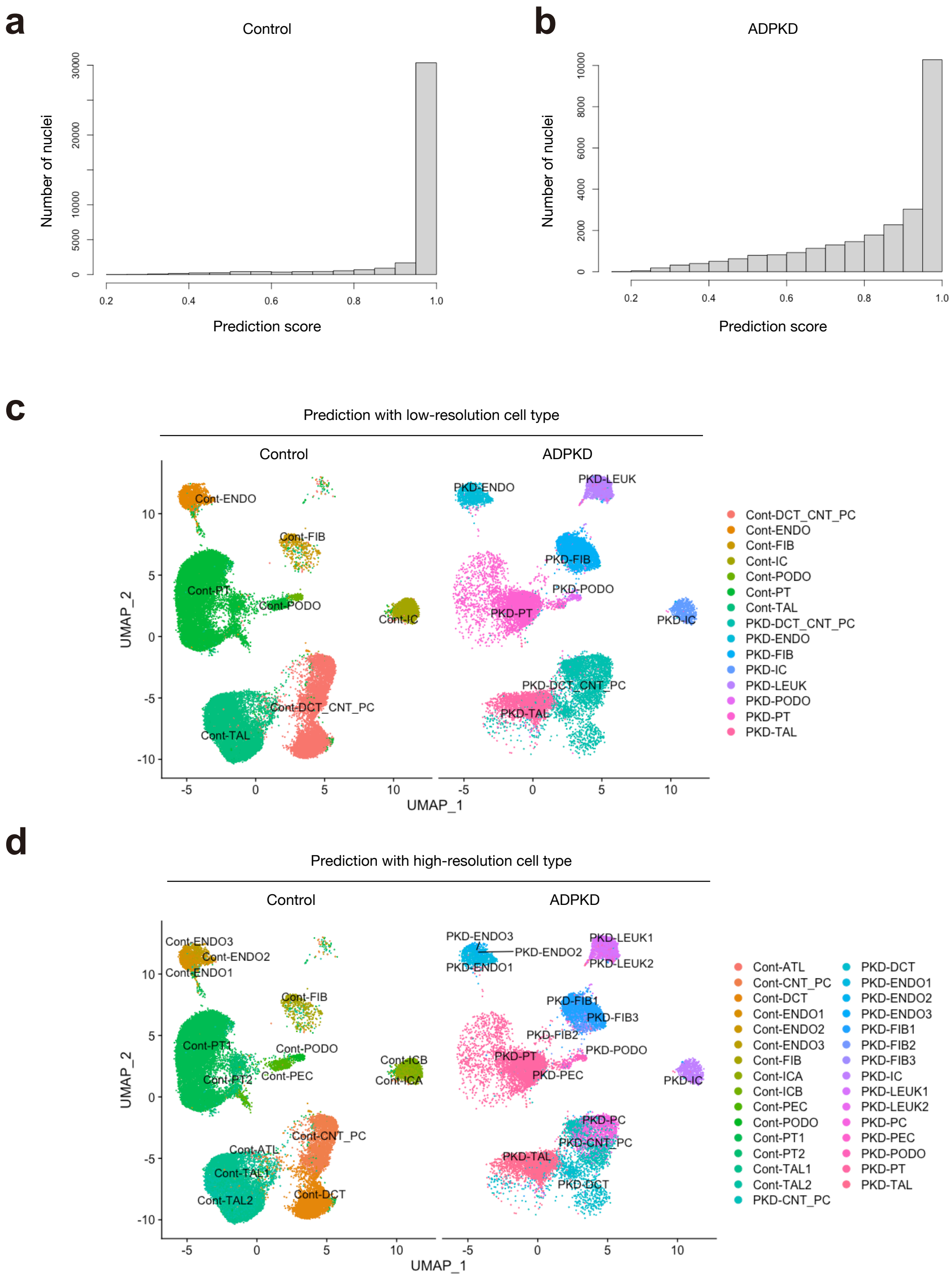

Supplementary Figure 7. Most of nuclei in ADPKD and control kidneys were predicted with high confidence

(**a**,**b**) Distribution of maximum prediction scores of nuclei calculated by the label transfer algorithm in Signac package for control (**a**) or ADPKD dataset (**b**). (**c**,**d**) UMAP plot of snATAC-seq dataset with predicted cell types through label transfer from snRNA-seq data with low-resolution cell types (**c**) or high-resolution cell types (**d**).

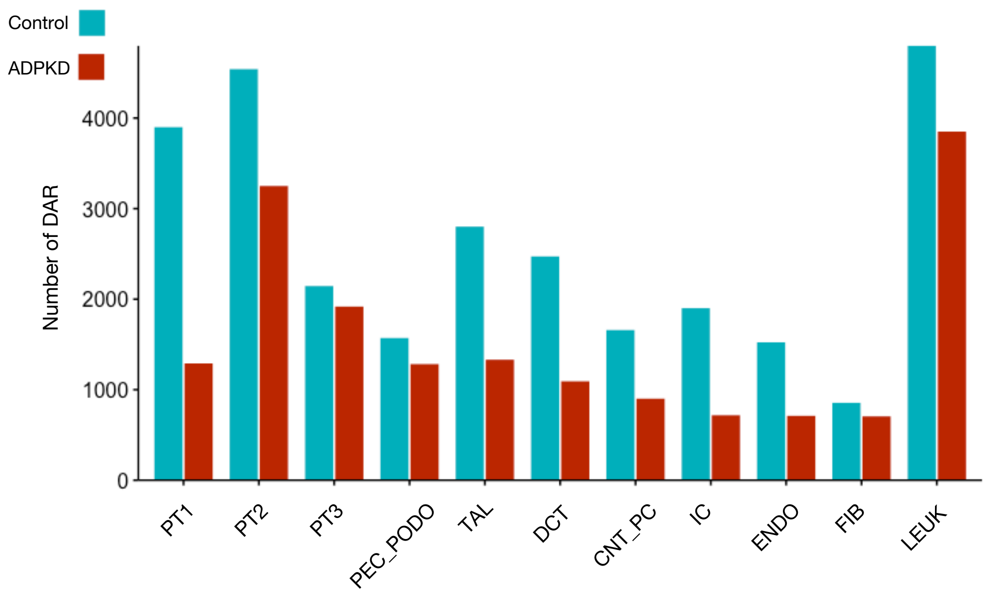

Supplementary Figure 8. Number of DAR in each cell type of control or ADPKD snATAC-seq dataset: Bar graph showing the difference of numbers of DAR between control (blue) and ADPKD (red). Source data are provided as a Source Data file.

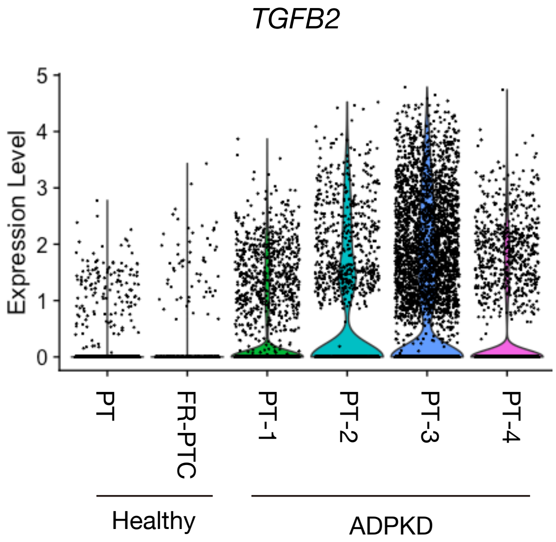

Supplementary Figure 9. *TGFB2* expression was upregulated PT lineage in ADPKD kidneys: A violin plot displaying *TGFB2* expression in PT subtypes among control or ADPKD kidney.

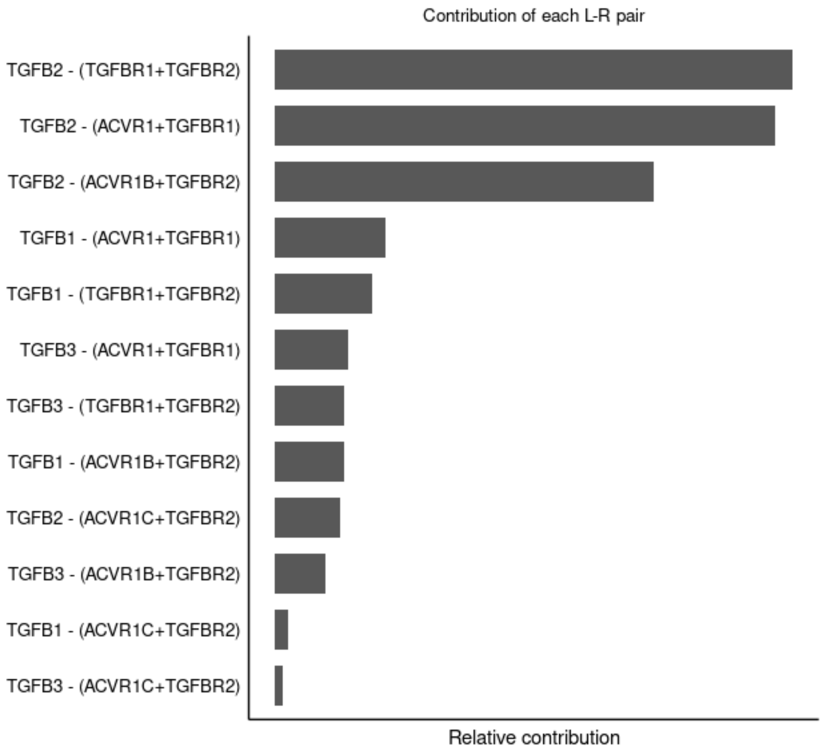

Supplementary Figure 10. Contribution of each ligand-receptor pair in TGFβ signaling: Bar plot showing relative contribution of each ligand-receptor pair in TGFβ signaling pathway.

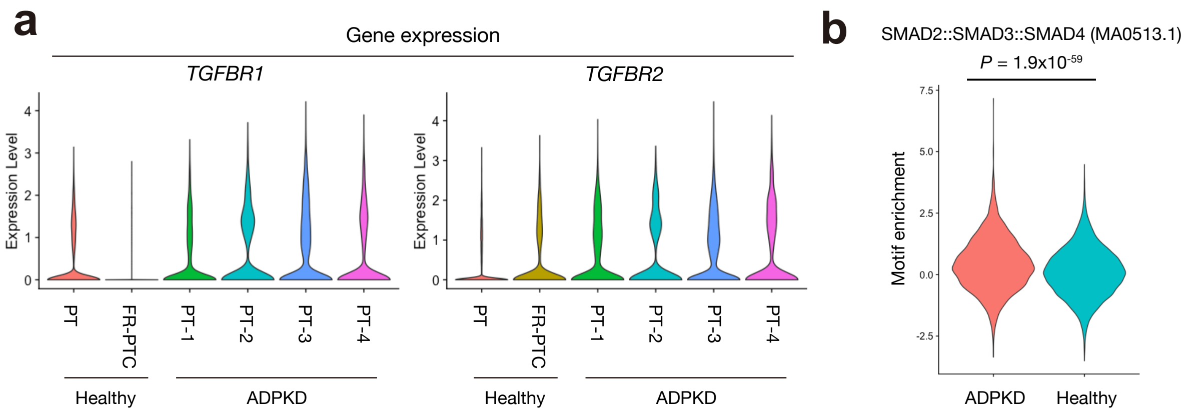

Supplementary Figure 11. Up-regulation of TGFβ receptors in FR-PTC

(**a**) Violin plot showing *TGFBR1* (left) or *TGFBR2* (right) gene expression among PT subtypes. (**b**) Violin plot showing SMAD2::SMAD3::SMAD4 binding motif (MA0513.1) enrichment score for PT lineage of ADPKD or control kidneys. Bonferroni adjusted p-values were used to determine significance.

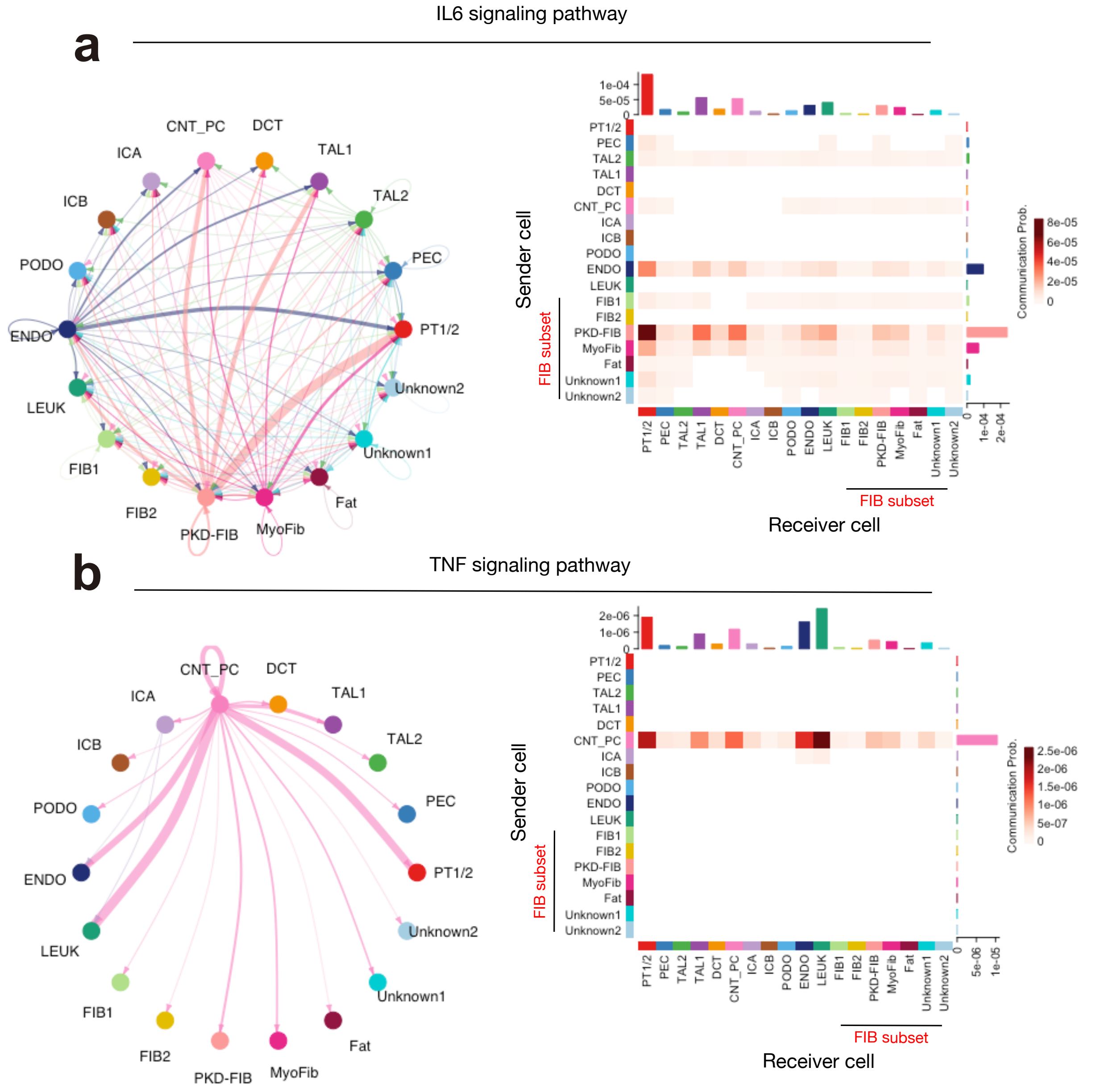

Supplementary Figure 12. Ligand-receptor analysis on all cell types and FIB subtypes for proinflammatory signaling pathways: Circle plot showing an inferred network (left) or heat map showing communication probabilities of signals from senders (secretors) to a receivers (targets) celltype (right) for IL6 signaling pathway (**a**) or TNF signaling pathway (**b**) among celltypes and FIB subtypes. Thickness of an arrow in a circle plot indicates interaction strength.

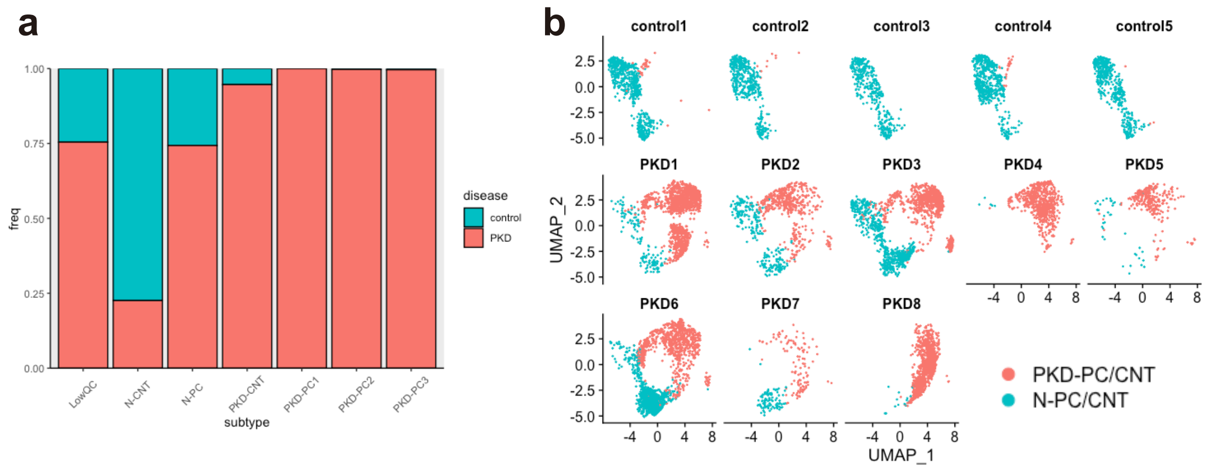

Supplementary Figure 13. Overlap between ADPKD and control cells in CNT_PC subclustering

(**a**) Proportion of disease group (control and ADPKD) in each subtype of subclustering on CNT_PC. (**b**) UMAP colored by subtypes, split by individual samples. PKD-PC/CNT: PKD-CDC1, 2, 3, and PKD-CNT. N-PC/CNT: N-PC and N-CNT. Source data are provided as a Source Data file.

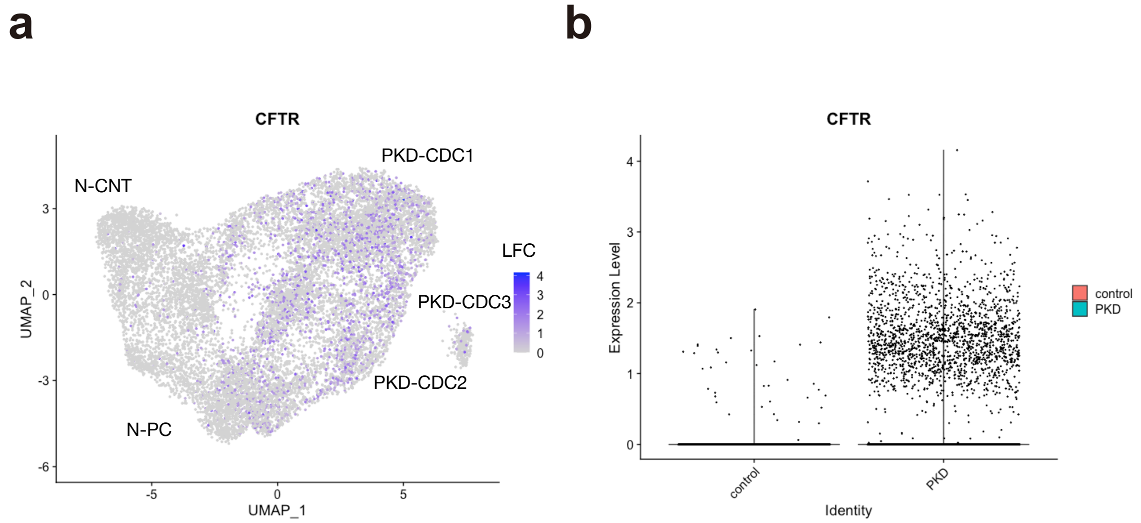

Supplementary Figure 14. ***CFTR*** **expressions in CNT_PC subclustering analysis**

(**a,b**) *CFTR* expression in CNT_PC subclusters displayed with UMAP plot (**a**) or violin plot comparing ADPKD and control cells (**b**). The color scale for each plot represents a normalized log-fold-change (LFC).

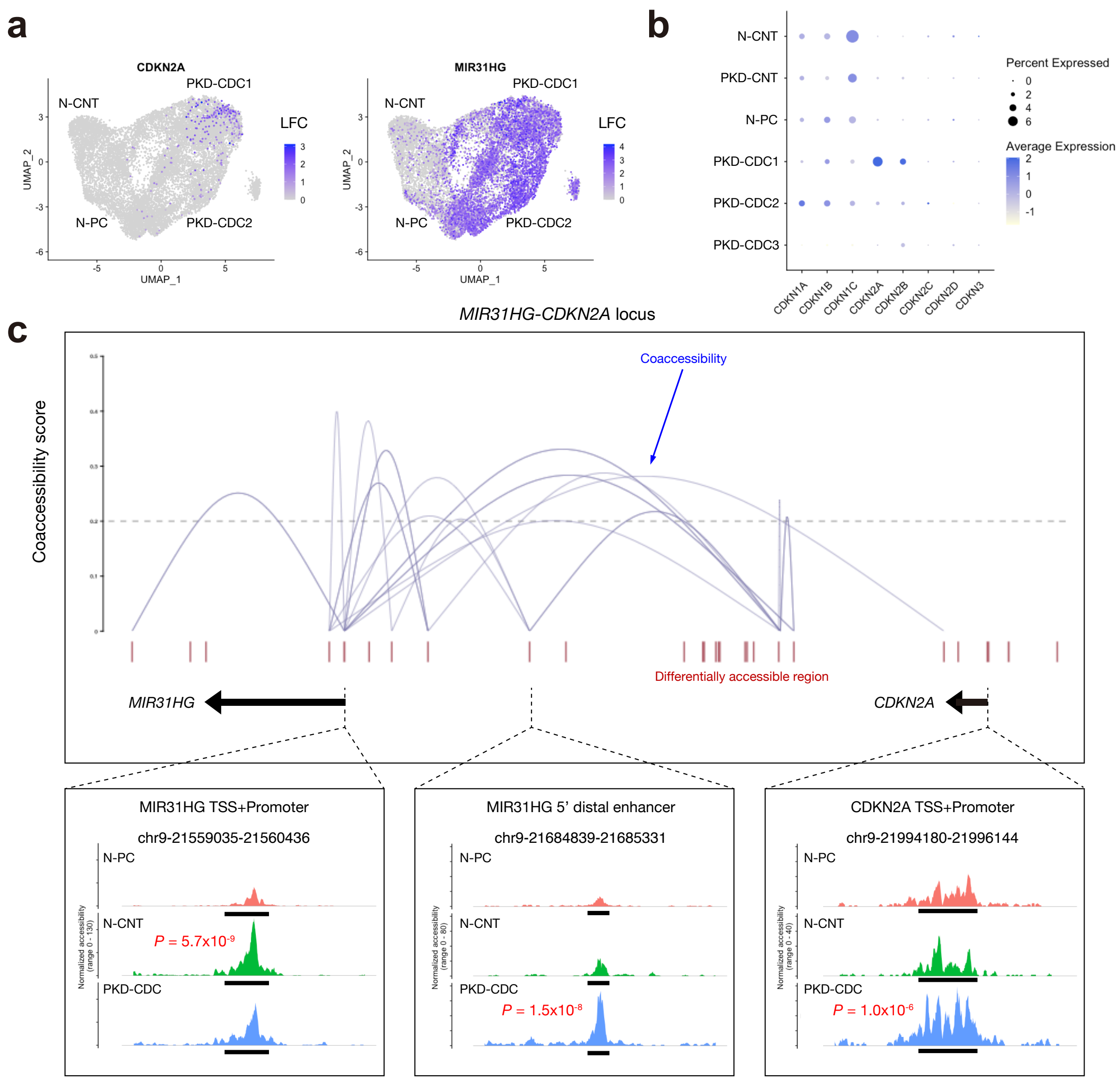

Supplementary Figure 15. Multimodal approach identified anti-senescence gene enhancer activation in PC subpopulations in ADPKD

(**a**) UMAP plot displaying *CDKN2A* (left) or *MIR31HG* (right) gene expression in the snRNA-seq. The color scale for each plot represents a normalized log-fold-change (LFC). (**b**) Dot plot showing gene expression patterns of the cyclin-dependent kinase inhibitor genes enriched in each of CNT_PC subpopulations. The diameter of the dot corresponds to the proportion of cells expressing the indicated gene and the density of the dot corresponds to average expression relative to all CNT_PC cells. (**c**) Cis-coaccessibility networks (CCAN, gray arcs) around the *MIR31HG* and *CDKN2A* gene in the ADPKD kidneys among accessible regions (red boxes) is shown (top). Fragment coverage (frequency of Tn5 insertion) around TSS (chr9:21559035-21560436) or 5' distal DAR (chr9:21684839-21685331) of *MIR31HG* and TSS of *CDKN2A* (chr9:21994180-21996144) are also shown (bottom, peak +/-3 Kb). Bonferroni adjusted p-values were used to determine significance for differential accessibility.

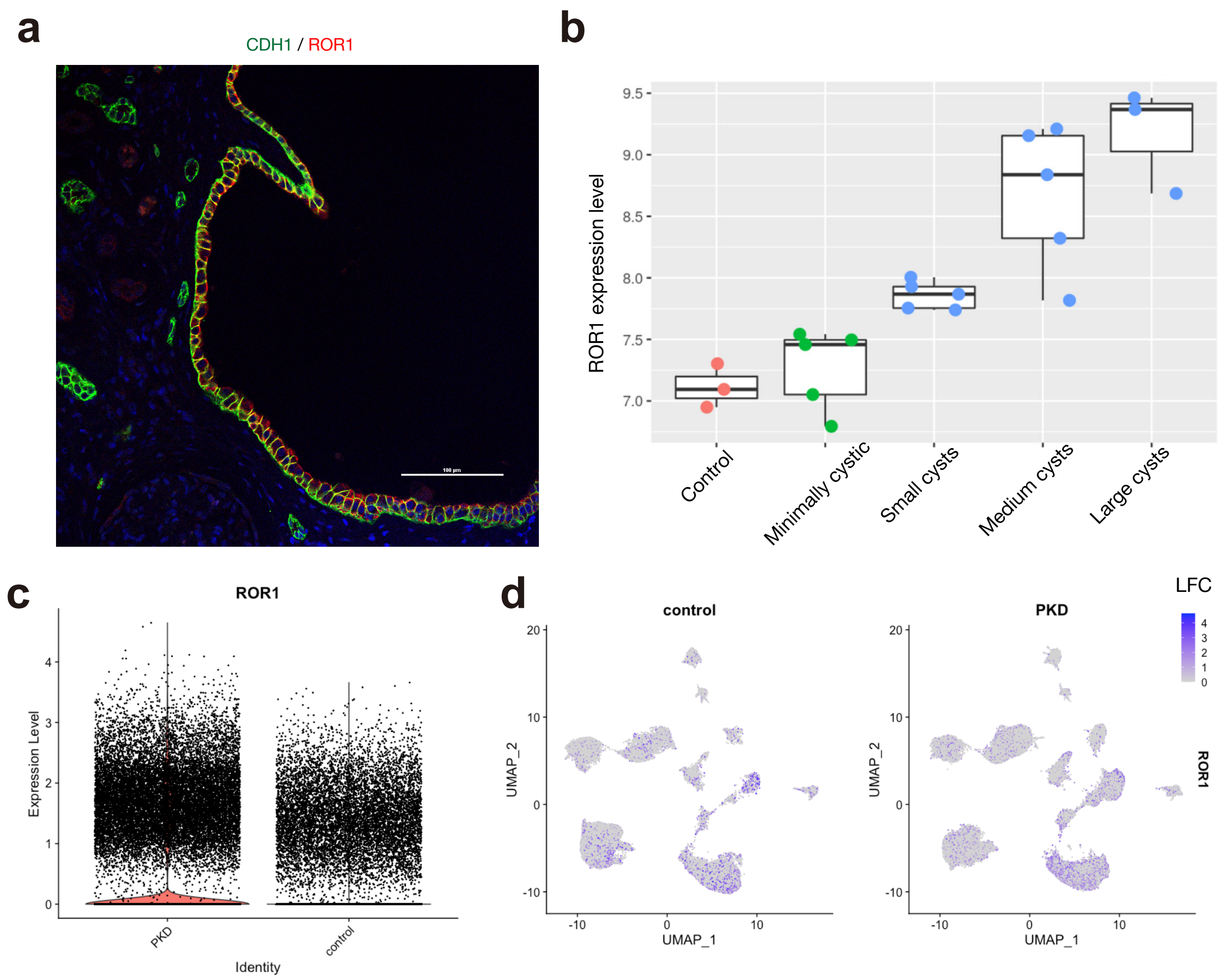

Supplementary Figure 16. ROR1 expression in PC lineages of ADPKD kidneys

(**a**) Representative immunohistochemical images of ROR1 (red) and CDH1 (green) in the ADPKD kidneys. Scale bar indicates 100 µm. (**b**) Correlation between *ROR1* expression and cyst size (GSE7869). Each dot represents a biological replicate for Control (n=3), Minimally cystic (n=5), Small cysts (n=5), Medium cysts (n=5) or Large cysts (n=3) of ADPKD patients. The data are presented as box-and-whisker plots depicting the median, quartiles and ranges. Source data are provided as a Source Data file. (**c**) Violin plot showing *ROR1* gene expression in ADPKD or control nuclei in all cell types. (**d**) UMAP plot displaying *ROR1* gene expression in the control dataset (left) or ADPKD dataset (right). The color scale for each plot represents a normalized log-fold-change (LFC).

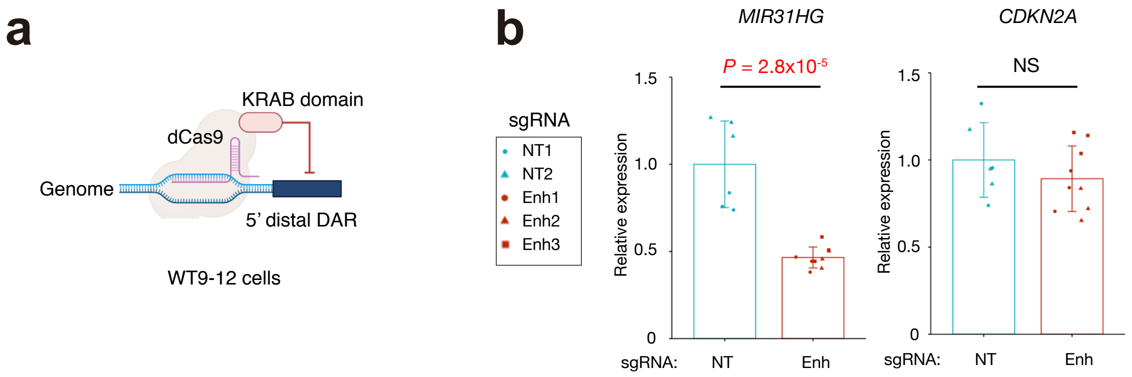

Supplementary Figure 17. CRISPR interference on *MIR31HG* enhancer in cyst cell line

(a) CRISPR interference targeting 5' distal DAR of *MIR31HG*. Schematic was created with BioRender. (b) RT and real-time PCR analysis of mRNAs for *GPRC5A* or *CDKN2A* in the WT9-12 cells with CRISPRi targeting the 5' distal potential enhancer (Enh) for *GPRC5A* gene. NT, non-targeting control. Each group consists of n = 6 (2 sgRNAs with 3 biological replicates for NT) or n=9 (3 sgRNAs with 3 biological replicates for Enh) data point. Bar graphs represent the mean and error bars are the s.d. Two-tailed Student's *t*-test. Source data are provided as a Source Data file.

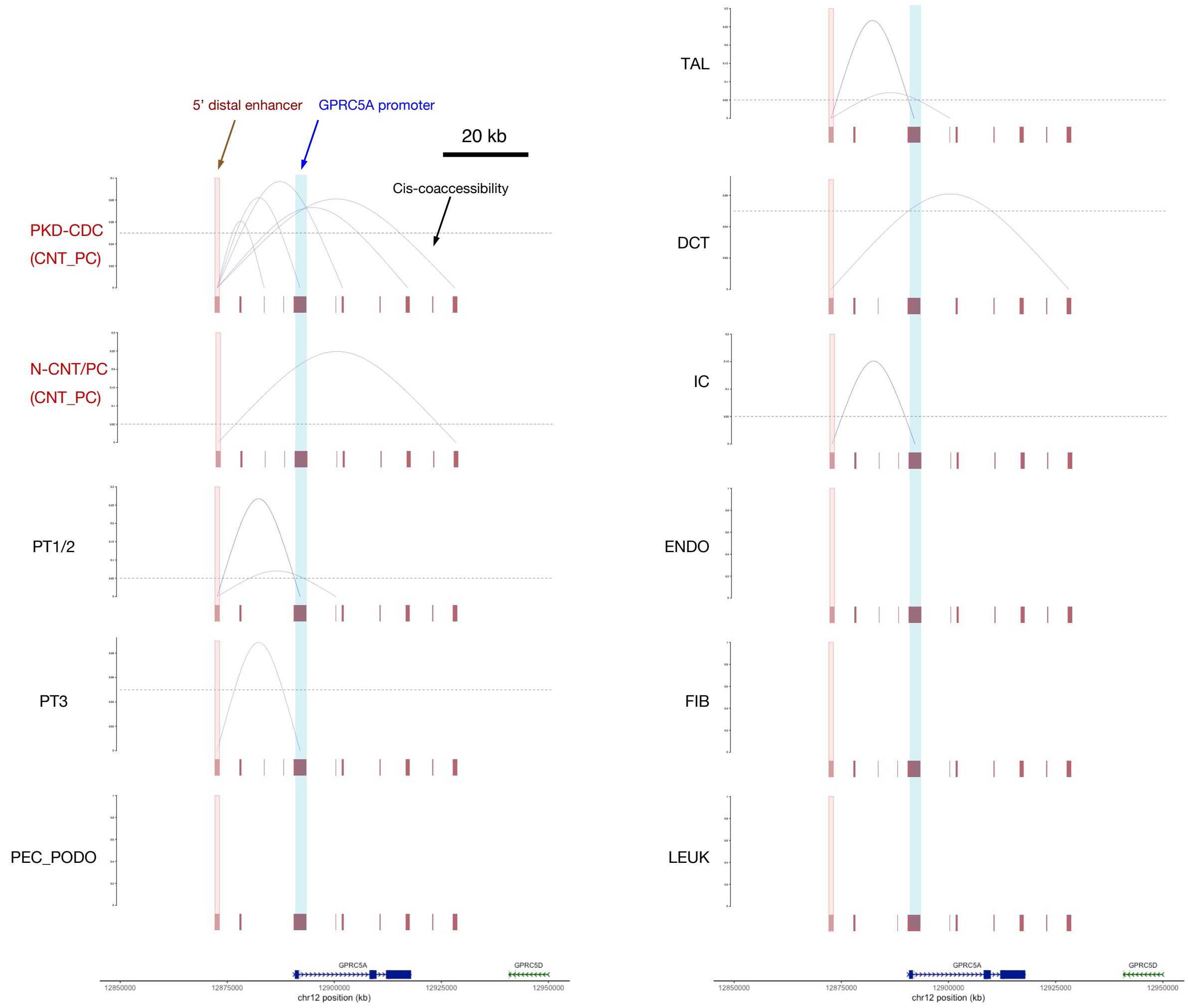

Supplementary Figure 18. CCAN around *GPRC5A* locus in each cell type in ADPKD kidneys: Cis-coaccessibility around 5' distal enhancer (red boxes) and TSS of *GPRC5A* (blue column) in each celltype is shown.

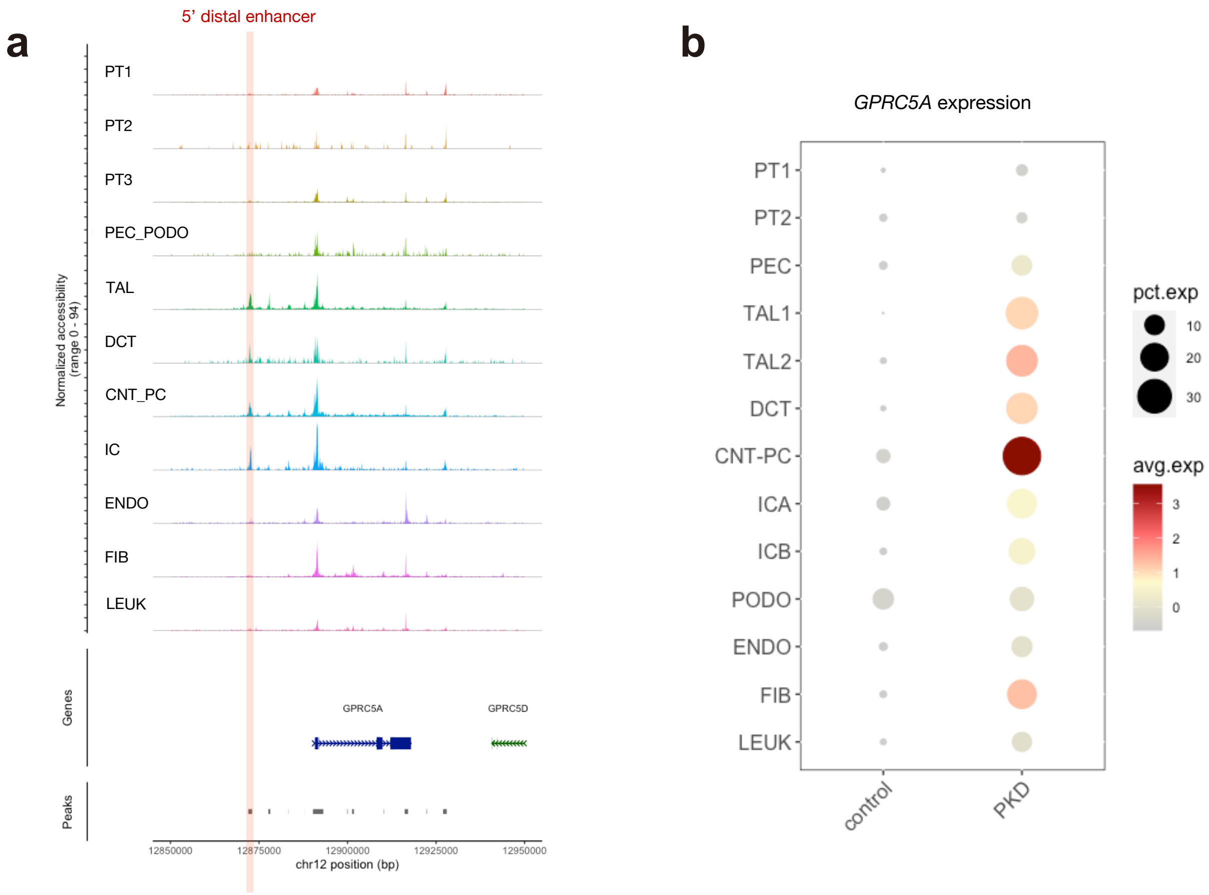

Supplementary Figure 19. Gene expression or chromatin accessibility of 5' distal enhancer for *GPRC5A* in each cell type of ADPKD kidneys: Accessibility on 5' distal enhancer of *GPRC5A* gene (red column, **a**) and dot plot showing GPRC5A expression in each celltype (**b**). The diameter of the dot corresponds to the proportion of cells expressing the indicated gene (pct. exp) and the density of the dot corresponds to average expression relative to all celltypes (avg. exp).

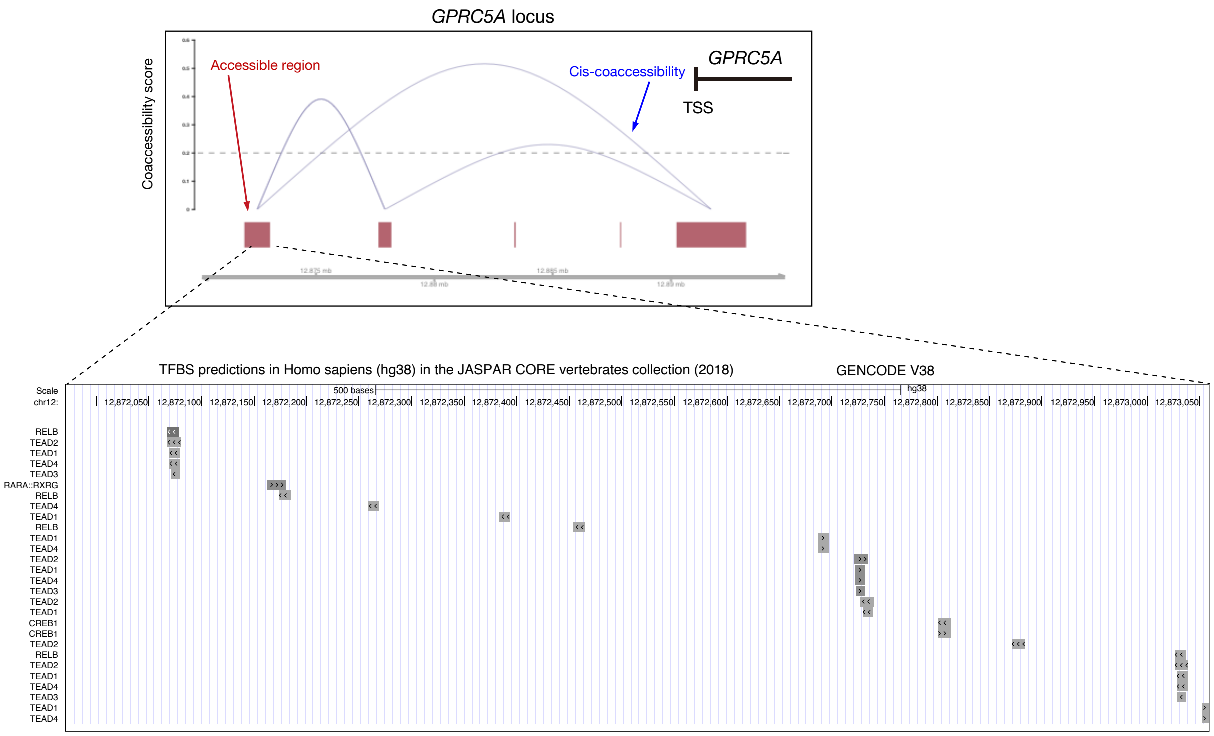

Supplementary Figure 20. Enrichment of NF-κB, TEAD, CREB and retinoic acid receptor family transcription factor binding motifs on 5' distal region of *GPRC5A* gene

The 5' distal region of *GPRC5A* gene (upper panel) has several binding motifs for cAMP responsive element binding protein 1 (CREB1) and retinoic acid receptors (RAR) as well as NF-κB (RELB) and TEAD family transcription factors (TEAD1-4), based on TFBS predictions in Homo sapiens (hg38) in the JASPAR CORE vertebrates collection (2018) shown on the UCSC genome browser (Enrichment score > 300, lower panel).

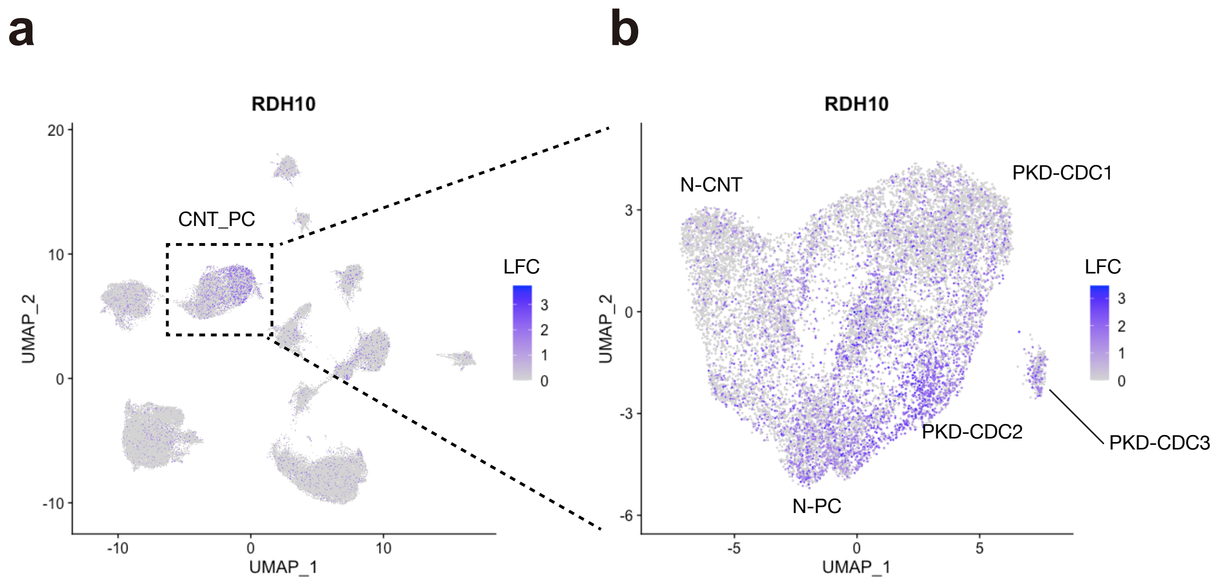

Supplementary Figure 21. *RDH10* expression in principal cell lineages in ADPKD kidneys

(**a**,**b**) UMAP plot displaying *RDH10* expression in the whole dataset (**a**) or CNT_PC subclusters (**b**). The color scale for each plot represents a normalized log-fold-change (LFC).

Sample ID	Gender	Age	eGFR (ml/min/1.73m^2)	Status prior to transplant	Duration of sample preservation (days)	Kidney size
PKD1	Female	50-60	7	Dialysis	810	1079 g
PKD2	Female	50-60	17	Pre-emptive	1207	803 g
PKD3	Female	50-60	17	Pre-emptive	216	1207 g
PKD4	Male	50-60	16	Pre-emptive	988	2061 g
PKD5	Female	60-70	23	Pre-emptive	535	1094 g
PKD6	Male	30-40	14	Pre-emptive	549	3170 g
PKD7	Male	40-50	10	Pre-emptive	629	2100 g
PKD8	Male	40-50	5	Pre-emptive	570	1531 g

Supplementary Table 1. Patient demographics and clinical information abstracted from the medical record

eGFR, estimated glomerular filtration rate. Status prior to transplant, whether the patient was on maintenance dialysis or not (pre-emptive transplantation). Duration of sample preservation, duration (days) between sample collection and snRNA-seq library preparation.

snRNA-seq	Control		ADPKD	
	Number	Frequency	Number	Frequency
PT1+PT2	10523	25.9%	12649	20.4%
PEC	869	2.1%	1634	2.6%
TAL1	13030	32.1%	11253	18.1%
TAL2	852	2.1%	1265	2.0%
DCT	6225	15.3%	4060	6.5%
CNT_PC	3747	9.2%	11143	18.0%
ICA	1096	2.7%	1900	3.1%
ICB	1014	2.5%	361	0.6%
PODO	624	1.5%	1585	2.6%
ENDO	1693	4.2%	3829	6.2%
FIB	625	1.5%	8244	13.3%
LEUK	339	0.8%	4064	6.5%
URO1	0	0.0%	65	0.1%
URO2	0	0.0%	21	0.0%
Total	40637	100%	62073	100%

Supplementary Table 2. The number or frequency of nuclei for each cell type quantitated in the whole filtered snRNA-seq dataset: PT, proximal tubule; PEC, parietal epithelial cells; TAL, thick ascending limb of Henle's loop; DCT, distal convoluted tubule; CNT_PC, connecting tubule and principle cells, ICA, Type A intercalated cells; ICB, Type B intercalated cells; PODO, podocyte; ENDO, endothelial cells; FIB, fibroblasts; LEUK, leukocytes; URO, uroepithelium.

snATAC-seq	Control		ADPKD	
	Number	Frequency	Number	Frequency
PT1	9176	27.3%	829	4.8%
PT2	3537	10.5%	96	0.6%
PT3	1198	3.6%	3119	18.0%
PEC_PODO	746	2.2%	292	1.7%
TAL	9289	27.6%	3406	19.6%
DCT	3095	9.2%	287	1.7%
CNT_PC	3036	9.0%	2765	15.9%
IC	1509	4.5%	615	3.5%
ENDO	1480	4.4%	1037	6.0%
FIB	498	1.5%	3241	18.7%
LEUK	57	0.2%	1678	9.7%
Total	33621	100.0%	17365	100.0%

Supplementary Table 3. The number or frequency of nuclei for each cell type quantitated in the whole filtered snATAC-seq dataset: PEC-PODO, parietal epithelial cells and podocyte; TAL, thick ascending limb; DCT, distal convoluted tubule; CNT_PC, connecting tubule and principle cells, ICA, Type A and TypeB intercalated cells; ENDO, endothelial cells; FIB, fibroblasts; LEUK, leukocytes.

Donor	Total RNA reads	RNA reads per cell	Total ATAC reads	ATAC fragments per cell
Control_1	243,702,726	27,226	343,687,555	13,892
Control _2	474,983,824	30,658	269,154,397	12,611
Control _3	212,996,173	22,783	396,525,222	17,493
Control _4	977,094,922	73,782	314,024,252	10,567
Control _5	132,744,441	13,696	267,097,032	10,168
ADPKD_1	297,480,919	24,616	324,886,233	9,755
ADPKD_2	460,253,422	31,274	372,923,215	39,809
ADPKD_3	370,314,010	31,171	374,516,045	26,190
ADPKD_4	334,024,895	22,458	253,540,663	9,233
ADPKD_5	303,934,085	28,312	282,728,864	11,359
ADPKD_6	422,011,072	25,609	348,954,290	27,627
ADPKD_7	307,102,696	93,429	518,627,214	34,513
ADPKD_8	372,678,869	101,520	201,042,995	14,884

Supplementary Table 4. The numbers of total reads and reads per nucleus in snRNA-seq and snATAC-seq data: Total reads and reads per cell in snRNA-seq and snATAC-seq data were shown.

Quality Control for snRNA libraries		
**Donor**	**Sequencing Saturation**	**Fraction reads with Valid Barcode**
Control_1	52.6	55.8
Control _2	49.7	64.7
Control _3	39.5	48.0
Control _4	65.7	51.8
Control _5	30.4	49.1
ADPKD_1	45.4	38.4
ADPKD_2	44.2	41.2
ADPKD_3	46.6	40.1
ADPKD_4	57.7	36
ADPKD_5	34.1	37.9
ADPKD_6	44.5	44.5
ADPKD_7	73.8	25.9
ADPKD_8	77.4	31.5

Supplementary Table 5 – Quality control for snRNA-seq libraries: The library complexity for the snRNA libraries was estimated with sequencing saturation for each donor. The fraction of read with a valid barcode in each donor.

**Quality Control for snATAC libraries**		
**Donor**	**Sequencing Saturation**	**Fraction reads with Valid Barcdode**
Control_1	36.2	98.3
Control _2	35.1	98.3
Control _3	37.1	98.3
Control _4	41.1	95.8
Control _5	37.3	95.8
ADPKD_1	25.6	95.4
ADPKD_2	40.5	97.1
ADPKD_3	31.9	97.7
ADPKD_4	17.4	95.8
ADPKD_5	32.7	95.8
ADPKD_6	27.1	96.9
ADPKD_7	49	97.2
ADPKD_8	28.4	85.8

Supplementary Table 6 – Quality control for snATAC-seq libraries: The sequencing saturation and the fraction of reads with a valid barcode in each donor were shown.

Sequence for sgRNA		
sgRNA name	sgRNA sequence	Targeted region (hg38)
GPRC5A-Enhancer #1	ACCTTCAGGGTCGCCTAACT	chr12:12872920-12872939
GPRC5A-Enhancer #2	TCTACCGGTTTATGTGTATA	chr12:12872506-12872525
GPRC5A-Promoter #1	TAAAGGCGGCCCTCGCCGGA	chr12:12891517-12891536
GPRC5A-Promoter #2	TCGGAGGAGTCCGATGCGCT	chr12:12891297-12891316
MIR31HG-Enhancer #1	AGTCATACACCTTGAATGGT	chr9:21684880-21684899
MIR31HG-Enhancer #2	CGGCAACAGCCCCTTAATGT	chr9:21685189-21685208
MIR31HG-Enhancer #3	CATATTTACTGTTAACACGA	chr9:21684789-21684808
Non-targeting #1	GGTAAGCGCGTGAGTCGAA	NA
Non-targeting #2	GAGGCGAGGTAAGACGCGG	NA

Supplementary Table 7 – Sequence for sgRNA expression
