# Supplementary material for: Defining cellular complexity in human autosomal dominant polycystic kidney disease by multimodal single cell analysis
Source: Nat Commun. 2022 Oct 30;13:6497. doi: 10.1038/s41467-022-34255-z (PMC9618568; doi:10.1038/s41467-022-34255-z)
Supplement: Supplementary file 3 — Description of Additional Supplementary Files [file 41467_2022_34255_MOESM3_ESM.pdf]

File Name: Supplementary Data 1

Description: Differentially expressed genes in each cell type in control kidneys

File Name: Supplementary Data 2

Description: Differentially expressed genes in each cell type in ADPKD kidneys

File Name: Supplementary Data 3

Description: Differentially expressed genes in ADPKD cells compared to control in each cell type

File Name: Supplementary Data 4

Description: Differentially accessible regions in each cell type in control kidneys

File Name: Supplementary Data 5

Description: Differentially accessible regions in each cell type in ADPKD kidneys

File Name: Supplementary Data 6

Description: Differentially accessible regions in ADPKD cells compared to control in each cell type

File Name: Supplementary Data 7

Description: Differentially activated genes in each cell type in control kidneys

File Name: Supplementary Data 8

Description: Differentially activated genes in each cell type in ADPKD kidneys

File Name: Supplementary Data 9

Description: Differentially activated genes in ADPKD cells compared to control in each cell type

File Name: Supplementary Data 10

Description: Differentially enriched chromVAR motifs in each cell type in control kidneys.

File Name: Supplementary Data 11

Description: Differentially enriched chromVAR motifs in each cell type in ADPKD kidneys.

File Name: Supplementary Data 12

Description: Differentially enriched chromVAR motifs in ADPKD cells compared to control in each cell type.

File Name: Supplementary Data 13

Description: Differentially expressed genes among PTC subtypes (snRNA-seq), related to Fig. 5

File Name: Supplementary Data 14

Description: Differentially expressed genes in each subtype in FIB subclustering analysis, related to Fig. 6

File Name: Supplementary Data 15

Description: Differentially expressed genes in each subtype in CNT_PC subclustering analysis (snRNA-seq), related to Fig. 7

File Name: Supplementary Data 16

Description: Differentially accessible regions of each subtype in CNT_PC subclustering analysis (snATAC-seq), related to Fig. 8

File Name: Supplementary Data 17

Description: Differentially enriched chromVAR motifs of each subtype in CNT_PC subclustering analysis (snATAC-seq), related to Fig. 8
