# Supplementary material for: Defining cellular complexity in human autosomal dominant polycystic kidney disease by multimodal single cell analysis
Source: Nat Commun. 2022 Oct 30;13:6497. doi: 10.1038/s41467-022-34255-z (PMC9618568; doi:10.1038/s41467-022-34255-z)
Supplement: Supplementary file 21 — Reporting Summary [file 41467_2022_34255_MOESM21_ESM.pdf]

nature portfolio

Corresponding author(s): Benjamin Humphreys

Last updated by author(s): 2022-9-19

Reporting Summary

Nature Portfolio wishes to improve the reproducibility of the work that we publish. This form provides structure for consistency and transparency in reporting. For further information on Nature Portfolio policies, see our Editorial Policies and the Editorial Policy Checklist.

Statistics

For all statistical analyses, confirm that the following items are present in the figure legend, table legend, main text, or Methods section.

n/a	Confirmed	
☐	☒	The exact sample size (*n*) for each experimental group/condition, given as a discrete number and unit of measurement
☐	☒	A statement on whether measurements were taken from distinct samples or whether the same sample was measured repeatedly
☐	☒	The statistical test(s) used AND whether they are one- or two-sided<br>*Only common tests should be described solely by name; describe more complex techniques in the Methods section.*
☐	☒	A description of all covariates tested
☐	☒	A description of any assumptions or corrections, such as tests of normality and adjustment for multiple comparisons
☐	☒	A full description of the statistical parameters including central tendency (e.g. means) or other basic estimates (e.g. regression coefficient) AND variation (e.g. standard deviation) or associated estimates of uncertainty (e.g. confidence intervals)
☐	☒	For null hypothesis testing, the test statistic (e.g. *F*, *t*, *r*) with confidence intervals, effect sizes, degrees of freedom and *P* value noted<br>*Give P values as exact values whenever suitable.*
☒	☐	For Bayesian analysis, information on the choice of priors and Markov chain Monte Carlo settings
☐	☒	For hierarchical and complex designs, identification of the appropriate level for tests and full reporting of outcomes
☒	☐	Estimates of effect sizes (e.g. Cohen's *d*, Pearson's *r*), indicating how they were calculated

Our web collection on statistics for biologists contains articles on many of the points above.

Software and code

Policy information about availability of computer code

Data collection	Mapping and generation of feature/peak-barcode matrix from fastq files was performed with the use of CellRanger (v6.0.0)/CellRangerATAC (v1.2.0).
Data analysis	Data analysis as described in Method section was performed in R (4.0.4) using the following software packages: Signac (v1.4.0); Seurat (v4.0.0); Monocle 3 (v1.0.0); Cicero (v1.3.5); chromVAR (v1.10.0); DoubletFinder (v2.0.3); Harmony (v1.0); CellChat (v1.1.3); CIBERSORTx (v1.0); pheatmap (1.0.12). No customized code was used for data analyses in this study. Analyses were following publicly available instructions from Seurat (http://satijalab.org/seurat/), Signac (https://satijalab.org/signac/), Cicero (https://cole-trapnell-lab.github.io/cicero-release/docs_m3/), CellChat (http://www.cellchat.org/) and CIBERSORTx (https://cibersortx.stanford.edu/).

For manuscripts utilizing custom algorithms or software that are central to the research but not yet described in published literature, software must be made available to editors and reviewers. We strongly encourage code deposition in a community repository (e.g. GitHub). See the Nature Portfolio guidelines for submitting code & software for further information.

Data

Policy information about availability of data

All manuscripts must include a data availability statement. This statement should provide the following information, where applicable:

- Accession codes, unique identifiers, or web links for publicly available datasets
- A description of any restrictions on data availability
- For clinical datasets or third party data, please ensure that the statement adheres to our policy

All relevant data are available from the corresponding authors on reasonable request. Sequencing data is deposited in GEO under accession number GSE185948. Previously published snATAC-seq data for five control kidneys are available in GEO (GSE151302). Gene expression, ATAC peaks and gene activities for each cell type

are also available via our interactive website; Kidney Interactive Transcriptomics (http://humphreyslab.com/SingleCell/). Public data repositories used for our analyses include Ensembl http://useast.ensembl.org., Genome UCSC browser http://genome.ucsc.edu., and JASPAR http://jaspar.genereg.net.

Field-specific reporting

Please select the one below that is the best fit for your research. If you are not sure, read the appropriate sections before making your selection.

- [x] Life sciences
- [ ] Behavioural & social sciences
- [ ] Ecological, evolutionary & environmental sciences

For a reference copy of the document with all sections, see nature.com/documents/nr-reporting-summary-flat.pdf

Life sciences study design

All studies must disclose on these points even when the disclosure is negative.

Sample size	No sample size calculation was performed. Samples from 8 ADPKD patients were processed to obtain ~8,000 single nuclei per sample. The number of nuclei analyzed in this study was sufficient to draw the conclusions described in the manuscript.
Data exclusions	For snATAC-seq, low-quality cells were removed from the aggregated snATAC-seq library peak region fragments < 1000, peak region fragments > 12000, %reads in peaks < 15, blacklist ratio > 0.005, nucleosome signal > 3 & TSS enrichment < 2) before normalization with term-frequency inverse-document-frequency (TFIDF). For snRNA-seq, we removed low-quality nuclei (nuclei with top 5% and bottom 1% in the distribution of feature count or RNA count, or those with %Mitochondrial genes > 0.25), and then we used DoubletFinder to remove heterotypic doublets (assuming 8% of barcodes represent doublets).
Replication	For snRNA-seq or snATAC-seq; samples from 8 ADPKD individuals were processed independently to generate libraries and similar results were obtained. Immunofluorescence studies (Fig.5) were performed on n = 3 samples, and similar results were obtained. Three independent experiments were performed for CRISPRi experiments / qPCR experiments, and similar results were obtained (Fig. 8g, i and Fig. S17)
Randomization	Randomization is not relevant to this study because there was a single experimental group with 8 samples.
Blinding	No blinding was used during data collection or analysis. It was not considered necessary for the study, because the measurements could not be affected by the operator.

Reporting for specific materials, systems and methods

We require information from authors about some types of materials, experimental systems and methods used in many studies. Here, indicate whether each material, system or method listed is relevant to your study. If you are not sure if a list item applies to your research, read the appropriate section before selecting a response.

Materials & experimental systems

n/a	Involved in the study	
[ ]	[x]	Antibodies
[ ]	[x]	Eukaryotic cell lines
[x]	[ ]	Palaeontology and archaeology
[x]	[ ]	Animals and other organisms
[ ]	[x]	Human research participants
[x]	[ ]	Clinical data
[x]	[ ]	Dual use research of concern

Methods

n/a	Involved in the study	
[x]	[ ]	ChIP-seq
[x]	[ ]	Flow cytometry
[x]	[ ]	MRI-based neuroimaging

Antibodies

Antibodies used

The following primary antibodies were used for immunofluorescence studies.
Host; Antigen; (Manufacturer; Catalogue number; Clone; Final dilution)
rabbit Anti-GPRC5A (Sigma; SAB4503536; 1:100)
goat Anti-ROR1 (Abcam; Ab111174; 1:125)
mouse Anti-E-Cadherin (BD Transduction; 610182; 36/E-Cadherin; 1:200)
rabbit Anti-VCAM1 (abcam; ab134047; EPR5047; 1:200)]
rabbit Anti-Cubilin (R&D Systems AF3700; 1:200)
rabbit Anti-LRP2 (Abcam, ab76969; 1:200)]

These sections were subsequently stained with the secondary antibodies listed below
Donkey Anti-Goat (Invitrogen; A11057; 1:200)
Donkey Anti-Mouse (Invitrogen; A21202; 1:200)
Donkey Anti-Rabbit (Invitrogen; A10042 or Jackson ImmunoResearch; 711-545-152; 1:200

Validation	The antibodies were validated by the manufacturer as below.<br>Antigen; Species; Application; Link<br>VCAM1 (mouse, rat, human; WB, IP, IHC-P, Flow Cyt, ICC/IF, ELISA; https://www.abcam.com/vcam1-antibody-epr5047-ab134047.html)<br>GPRC5A (human, rat, mouse; ELISA, western blot; https://www.sigmaaldrich.com/US/en/product/sigma/sab4503536)<br>ROR1 (human: IHC-P; https://www.abcam.com/ror1-antibody-ab111174.html)<br>E-Cadherin (Human, Mouse, Rat, Dog; WB, IF, IHC, Immunoprecipitation; https://www.bdbiosciences.com/en-eu/products/reagents/<br>Cubilin (Human, Mouse, Rat; ELISAs and Western blots; https://www.rndsystems.com/products/human-mouse-rat-cubilin-antibody_af3700)<br>LRP2 (Mouse, Rat, Human, Monkey; IHC-P; https://www.abcam.com/lrp2--megalin-antibody-ab76969.html?productWallTab=ShowAll)<br>microscopy-imaging-reagents/immunofluorescence-reagents/purified-mouse-anti-e-cadherin.610181)

Eukaryotic cell lines

Policy information about cell lines

Cell line source(s)	WT9-12cells were purchased from ATCC (https://www.atcc.org/products/crl-2833)
Authentication	Not authenticated by the authors.
Mycoplasma contamination	All cells were negative for Mycoplasma contamination.
Commonly misidentified lines<br>(See ICLAC register)	No commonly misidentified cell lines were used.

Human research participants

Policy information about studies involving human research participants

Population characteristics	Supplemental Table 1 lists the metadata for the patients from which the data were generated. There was no genotype information available from the participants.
Recruitment	8 human kidney tissues from nephrectomy were used with informed consent.
Ethics oversight	ADPKD kidney cortical cup samples were obtained from patients undergoing simultaneous native nephrectomy and living donor kidney transplantation at the University of Maryland Medical Center (Baltimore, MD). All participants provided written informed consent for participation and tissue donation. The human subjects protocol was approved by the Institutional Review Board of the University of Maryland, Baltimore.

Note that full information on the approval of the study protocol must also be provided in the manuscript.
